# Multi-Generational Kinship, Multiple Mating, and Flexible Modes of Parental Care in a Breeding Population of the Veery (*Catharus fuscescens*), a Trans-Hemispheric Migratory Songbird

**DOI:** 10.1371/journal.pone.0157051

**Published:** 2016-06-22

**Authors:** Matthew R. Halley, Christopher M. Heckscher, Venugopal Kalavacharla

**Affiliations:** 1 Department of Agriculture and Natural Resources, Delaware State University, Dover, Delaware, United States of America; 2 Center for Integrated Biological and Environmental Research, Delaware State University, Dover, Delaware, United States of America; University of Missouri, UNITED STATES

## Abstract

We discovered variable modes of parental care in a breeding population of color-banded Veeries (*Catharus fuscescens*), a Nearctic-Neotropical migratory songbird, long thought to be socially monogamous, and performed a multi-locus DNA microsatellite analysis to estimate parentage and kinship in a sample of 37 adults and 21 offspring. We detected multiple mating in both sexes, and four modes of parental care that varied in frequency within and between years including multiple male feeders at some nests, and males attending multiple nests in the same season, each with a different female. Unlike other polygynandrous systems, genetic evidence indicates that multi-generational patterns of kinship occur among adult Veeries at our study site, and this was corroborated by the capture of an adult male in 2013 that had been banded as a nestling in 2011 at a nest attended by multiple male feeders. All genotyped adults (*n* = 37) were related to at least one other bird in the sample at the cousin level or greater (*r* ≥ 0.125), and 81% were related to at least one other bird at the half-sibling level or greater (*r* ≥ 0.25, range 0.25–0.60). Although our sample size is small, it appears that the kin structure is maintained by natal philopatry in both sexes, and that Veeries avoid mating with close genetic kin. At nests where all adult feeders were genotyped (*n* = 9), the male(s) were unrelated to the female (mean *r* = -0.11 ± 0.15), whereas genetic data suggest close kinship (*r* = 0.254) between two male co-feeders at the nests of two females in 2011, and among three of four females that were mated to the same polygynous male in 2012. To our knowledge, this is the first evidence of polygynandry occurring among multiple generations of close genetic kin on the breeding ground of a Nearctic-Neotropical migratory songbird.

## Introduction

A primary goal of evolutionary biology is to explain the proximate and ultimate causes of mating system diversity [1,2]. In diploid animals that reproduce sexually, optimal mating strategies are expected to differ between the sexes because the cost of gamete production is much lower for males than females [3]. Variability in mating and parental behavior is thought to be an outcome of reproductive conflicts unfolding in ecologically and socially stochastic environments [4,5]. In birds, males that monopolize multiple females by physically preventing them from copulating with other males (female defense polygyny), and/or by guarding a resource that they depend on (resource defense polygyny), can greatly increase their annual reproductive output, whereas the clutch size of the female is ecologically and physiologically constrained [4,6]. Polygyny is therefore expected to be the default strategy of males, unless they are thwarted by social and/or environmental conditions—monogamy represents a less-than-ideal scenario for males, relative to polygyny. In this context, multiple mating may be a counter strategy by which females mated to polygynous males mitigate the risk of mate desertion [3], augment parental support [7,8], minimize reproductive failure because of genetic incompatibility or semen storage failure [9], and/or produce genetically diverse offspring [10], among other hypotheses.

Species that exhibit cooperative parental care (i.e., more than two adults attending a single brood) are of special interest to sociobiologists because of the apparent altruistic behavior of non-breeding adults [11–13]. In such cases, cooperative parental care is inferred to be expressed when kinship is greater within than between groups, a result of some individuals delaying or foregoing natal dispersal, and helping behavior by non-breeding genetic relatives is subsequently favored by kin selection for its inclusive benefit [14]. The frequency of cooperative parental care may be inversely related to the availability of a scarce resource—a proximate cause [1]—like nest cavities in Red-cockaded Woodpeckers (*Picoides borealis*), but even in that case the ultimate (functional) explanation of the helping behavior by non-breeders is inferred to be the kin selected benefit [15]. A majority of cooperatively breeding bird species live in socially cohesive family groups, lending support to this theory [16,17].

However, in some species cooperative parental care has apparently evolved among non-relatives, and multiple males in breeding condition feed at the nest of a single female [18,19]. These species have generally been excluded from comparative studies focused on altruistic behavior, because all male feeders have a potential stake in paternity and thus a direct benefit—kin selection is not expected to play a role in these systems [20]. Davies [21] proposed an ecological explanation for variable modes of parental care in a non-migratory population of the Dunnock (*Prunella modularis*), in which close genetic kinship was not detected among adults, and modes of parental care, including cooperative parental care, fluctuated within and between years. Like Red-cockaded Woodpeckers [15], the frequency of cooperative parental care at Dunnock nests was found to be inversely correlated with a limiting resource—food availability [21]. When widespread cooperative parental care was discovered in the migratory Bicknell's Thrush (*Catharus bicknelli*) [19], a species for which there is no evidence that closely related adults interact on the breeding grounds [22] (but see [23]), it was generally assumed that the behavior was derived, having evolved under ecological constraints endemic to the species's harsh montane breeding habitat. That hypothesis was supported by the apparent lack of cooperative parental care in other *Catharus* species, and evidence that, like the Dunnock [21], the frequency of cooperative parental care in Bicknell's Thrush is inversely related to prey availability [24].

We discovered cooperative parental care in the Veery (*Catharus fuscescens*), a close relative of Bicknell's Thrush that breeds at lower elevations, in forests where invertebrate prey are presumably abundant [25]. Veeries have a complex annual cycle that includes two trans-hemispheric migratory events and a synchronized intratropical migration within South America [26–28]. They breed in temperate deciduous forests in North America, where adults undergo a complete molt at the end of the Nearctic summer before migrating to South America. The sexes are monomorphic and cannot be distinguished by sight unless individuals are marked by researchers (color-banded). Notwithstanding, early investigators of unbanded Veeries frequently attributed behavioral observations to each sex as if they were distinguishable [29–31], a practice that contributed to the impression that social monogamy was ubiquitous in the species [32].

We quantified the social and genetic mating systems of a color-banded breeding population of Veeries in northern Delaware, USA, via digital video and direct observation of nesting activities and a multi-locus genetic analysis of 37 adults and 21 offspring. To test whether kin selection could explain the cooperative parental care, we examined pairwise estimates of genetic relatedness among adults in relation to patterns of nest attendance [33]. Cooperative parental care is apparently rare among migratory species and therefore has been interpreted by some authors to be evidence that migration has an entropic effect on kin structure via facilitating natal dispersal [34–37]. We decided *a priori* that if close kinship (*r* ≥ 0.25) was not detected among and between second-year (SY) and after-second-year (ASY) adults at the study site, or if kinship was rare, then kin selection would not be supported. We also estimated genetic parentage at a sample of Veery nests via parental exclusion [38] to determine whether males and females mated with multiple partners (i.e., multiple broods per sire, and multiple sires per brood, respectively).

## Methods

### Data Collection

We conducted fieldwork at White Clay Creek State Park (hereafter White Clay), New Castle County, Delaware (39° 44' N, 75° 45' W), during May, June, and July of 2011 and 2012. CMH has monitored the breeding activities of color-banded Veeries at White Clay since 1998. We located nests (*n* = 36) by following adults carrying nest material or food, and subsequently monitored them via direct observation or with a digital video camera (Oregon Scientific ATC2K, Tulatin, OR, USA), which was placed ~0.5 m from the nest rim and concealed in vegetation. The close placement of the cameras enabled identification of all banded individuals that visited each nest. Nests were checked at ≤ 3 day intervals to change camera batteries and retrieve video footage.

We captured Veeries in mist nets and fitted each bird with a unique combination of three colored plastic leg bands and a numbered aluminum band (Bird Banding Laboratory, USGS Patuxent Wildlife Research Center, Patuxent, Maryland, USA). Individuals were thereafter identified by four letter codes corresponding to various color combinations (e.g., AXRO, FLOX). We determined sex via the presence or absence of a cloacal protuberance or brood patch, and assigned each bird to an age-class (SY or ASY) upon examination of the shape and size of the reduced outer primary (p10), presence/absence of "buffy tips" on the outer greater coverts, and shape of the rectrices [39]. In 2011, we banded 34 nestlings from 10 broods. In 2012, we collected a small blood sample (≤ 150μL) from the brachial vein of 37 adults (14 females, 23 males), and 16 nestlings (5–9 days after hatching). Each blood sample was transferred to a vial containing 1.0 mL Queen's Lysis Buffer and stored at 4°C until DNA extraction [40]. Dead nestlings (*n* = 3) and unhatched eggs (*n* = 2) were collected from 3 broods and frozen for use in genetic analysis. No adverse effects of blood collection were observed in adults and nestlings. Adults were released immediately following blood collection, and no infections or abnormalities were observed in birds that were recaptured several days later. Nestlings resumed begging shortly after being returned to the nest after blood collection.

All field research activities (e.g., mist netting, banding, blood collection) were reviewed and specifically approved by the State of Delaware Department of Natural Resources & Environmental Control, Division of Parks and Recreation. Bird banding and blood collection was conducted under US Federal Bird Banding and Marking Permit #23711, granted to the second author (CMH). Our research did not involve an endangered or protected species, nor did it involve any captive animals. To our knowledge, no animals were physically harmed because of our research activities. Our protocol for collecting blood (brachial venipuncture) is used widely among ornithologists, and MRH was experienced with the procedures before initiating the study.

### Video Review

We used Windows Movie Maker 6.0 (Microsoft Corporation, Redmond, WA) to identify adult feeders in 57 video sessions from 10 nests (mean duration ± SD = 2.69 ± 1.03 h). Nests were recorded for an average of 15.67 ± 10.76 h (range 6.78–37.73 h). Videos of known second or third nesting attempts (i.e., following failure of the first brood) were removed from the sample. We then followed previous studies of polygynandrous species in treating each nest as if it were a first attempt [19,41]. We included nests for which the identities of multiple male feeders were confirmed by direct observation but not captured on video, and excluded nests that were attended by an unbanded adult.

### Genetic Analysis

We used DNA Mini Kits (Qiagen, Inc., Valencia, CA) to isolate DNA from whole blood and tissue samples. We amplified six microsatellite markers (Cuμ02, Cuμ04, Cuμ05, Cuμ10, Cuμ28, Cuμ32) that had been previously identified in the genome of congeneric Swainson's Thrush (*Catharus ustulatus*) [42], via polymerase chain reactions (PCR) with three labeled fluorescent primers (6FAM, VIC, NED, Life Technologies Corporation, Chicago, IL) and equivalent thermocycling conditions: 30 cycles; 94°C for 45 sec, 53°C for 45 sec, 72°C for 45 sec. We electrophoresed each PCR product with positive and negative controls and a 100bp DNA size standard in 2% agarose gels stained with ethidium bromide, and visually checked the polymorphism of each locus on silver-stained 6% denaturing polyacrylamide gels (55W for 2 h) with a 10bp single-stranded reference ladder. We multiplexed the samples (3 loci per tube) and submitted them to the Delaware Biotechnology Institute (Newark, DE), where fragments were sized in a 3130xl Genetic Analyzer (Applied Biosystems, Inc.) with a GeneScan 500LIZ size standard.

We generated a multi-locus genotype for each bird following standard procedures [38]. PEAKSCANNER 1.0 (Applied Biosystems) was used to score fragment sizes, Minitab 16 (Microsoft Corporation) to plot allele frequencies for each locus, and CERVUS 3.0 to analyze allele frequencies in the dataset [43]. Eighty alleles were found in the sample and all six markers showed high levels of heterozygosity (mean = 0.82, SD = 0.09) and polymorphic information content (mean = 0.82, SD = 0.18; Table 1). No significant deviations from Hardy-Weinberg equilibrium were detected. We calculated parental exclusion probabilities with CERVUS 3.0 and assigned parentage based on statistical confidence [43]. First, we compared offspring alleles to those of their social mother and assigned maternity if the mother possessed either offspring allele at each locus. The remaining paternal-obligate allele was then tested against a pool of potential fathers. We followed previous authors in attributing a mismatch at a single locus to mutation, typing error, or presence of a null allele, and did not exclude parentage in those cases [19,38,43].

**Table 1:** Allele frequency and polymorphism data for six microsatellite markers amplified from 37 adult Veeries (*Catharus fuscescens*) and 21 offspring at White Clay Creek State Park, New Castle County, Delaware. Data were calculated via CERVUS 3.0.

Locus	k^a^	f^b^	H_E_^c^	H_O_^d^	PIC^e^	NE1^f^	NE2^g^
Cuμ02	16	0.121	0.925	0.983	0.911	0.288	0.168
Cuμ04	11	0.290	0.836	0.860	0.810	0.499	0.330
Cuμ05	14	0.500	0.713	0.724	0.684	0.671	0.482
Cuμ10	14	0.207	0.900	0.948	0.884	0.353	0.214
Cuμ28	18	0.181	0.898	0.914	0.880	0.363	0.221
Cuμ32	7	0.422	0.762	0.517	0.729	0.622	0.438

^a^ Number of alleles

^b^ Frequency of the most common allele

^c^ Expected level of heterozygosity

^d^ Observed level of heterozygosity

^e^ Polymorphic information content

^f^ Mean non-exclusion probability with information on one known parent (i.e., probability of not excluding a single candidate parent with knowledge of one parental genotype)

^g^ Mean non-exclusion probability without the genotype of one parent

We used KINGROUP 2.0 to calculate Queller's *r* with bias correction [33] for each unique pair of adults of known sex and age-class (*n* = 666 combinations). Relatedness (*r*) is a continuous measure of identity by descent that allows for categorical discernment of full siblings and parents/offspring (*r* = 0.5), half siblings, uncles/nephews, grandparents/grandchildren (*r* = 0.25), cousins (*r* = 0.125), and unrelated individuals (*r* ≤ 0). This method was validated in our data by *r*-values of known parent-offspring pairs (*n* = 18, mean = 0.44, SE = 0.03) being comparable to expected values (*r* = 0.50).

### Statistical Analysis

We used Minitab 16 (Microsoft Corporation) to perform a cluster analysis of the entire dataset using correlation distance measures and the 'complete linkage' method, which bases inter-cluster distances for clusters with multiple observations on the maximum distance between an observation in one cluster and an observation from the other. Relatedness (*r*) data were non-normally distributed, so we used a Mann-Whitney *U*-test to determine if *r*-values differed between within-cluster and between-cluster genotype pairings. All means are reported as ± 1 SD.

## Results

### Kin Structure

**Fig 1:**
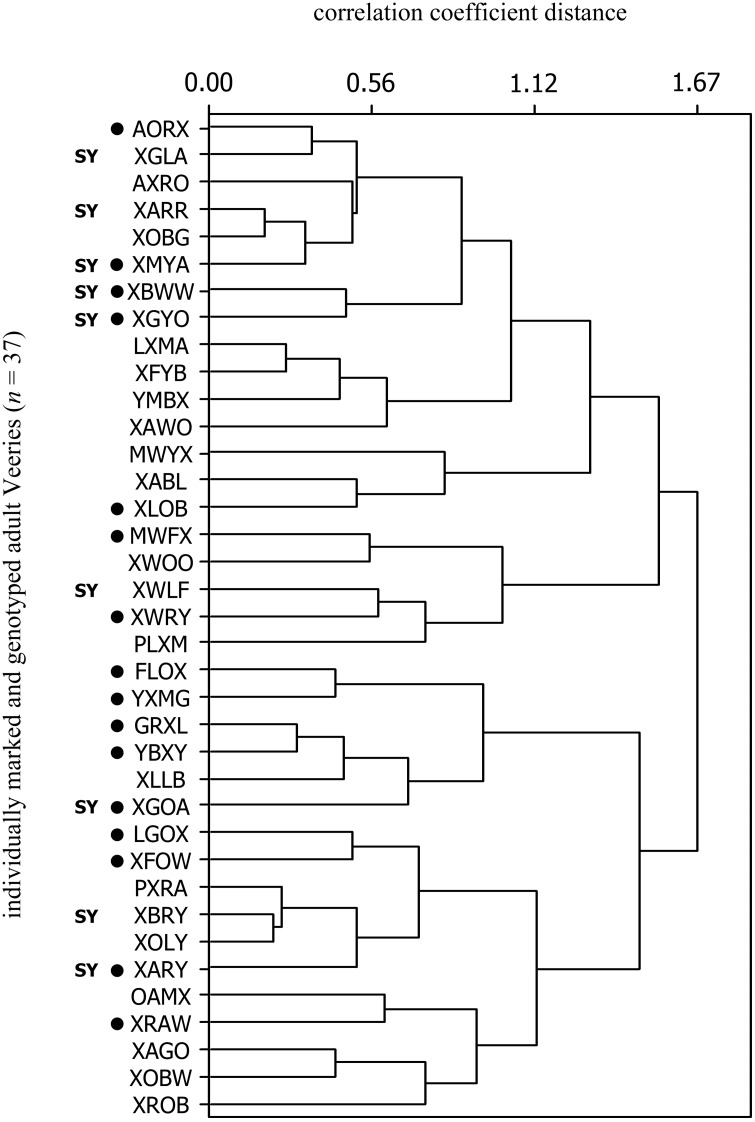
Dendrogram showing results of cluster analysis of pairwise *r*-values (*n* = 666) from 37 adult Veeries (16 females, 21 males, denoted by four-letter color combinations; see text) sampled from a population in White Clay Creek State Park, New Castle County, Delaware. Tree was constructed with a 'complete linkage' clustering method. Females are denoted by black circles, and second-year birds are marked 'SY'. The dendrogram does not represent genealogical relationships, nor do clusters imply a persistent social cohesiveness within the population.

**Fig 2:**
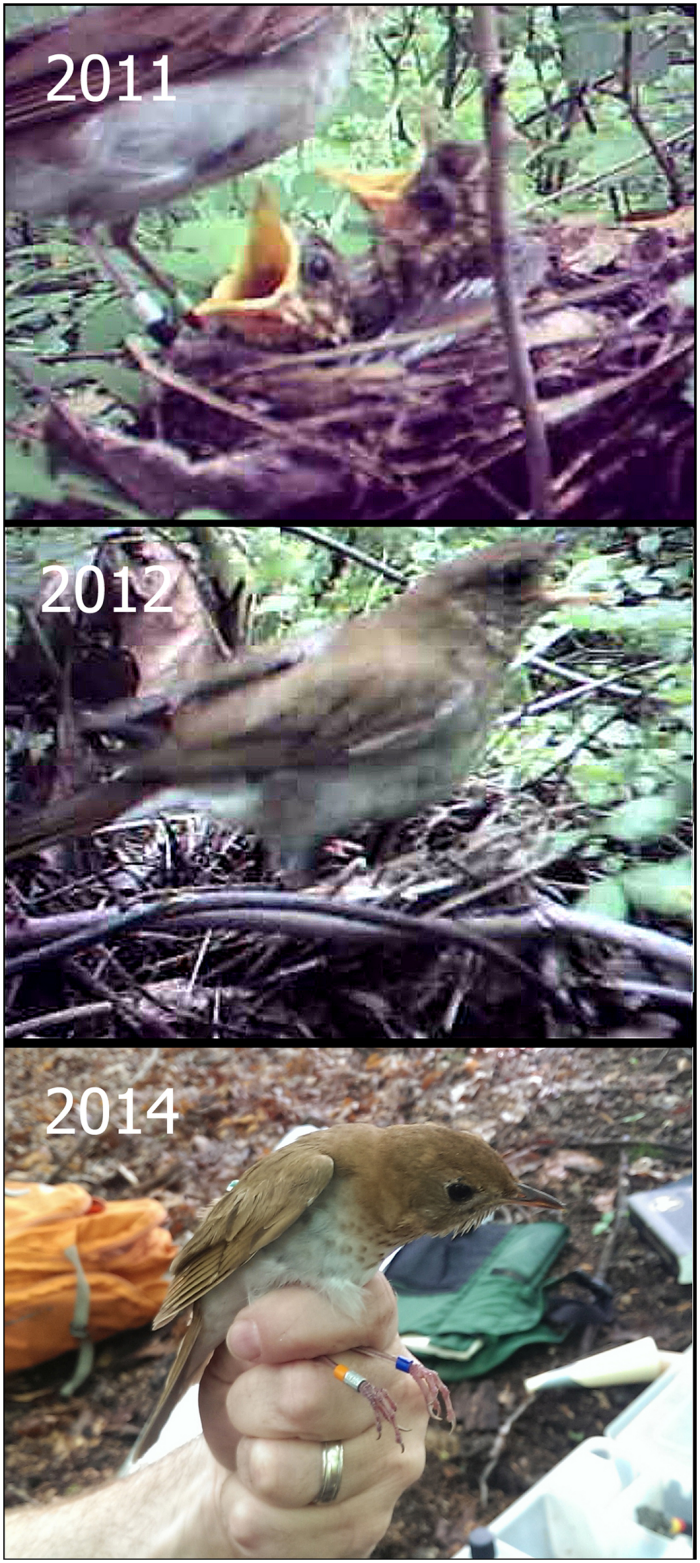
(2011) Still image from digital video showing female GRXL during a food delivery to nestlings B/OX and B/WX on June 11, 2011. This nest was also provisioned by two closely related males (AXRO and LXMA, *r* = 0.254). (2012) Still image from video showing GRXL in agonistic posture (i.e., "horizontal stretch" with bill gaping) [44] immediately prior to the arrival of AXRO (see text for context). (2014) Digital photograph of B/OX as an adult male, 22 May 2014 (photo by M. Gutierrez-Ramirez). As an adult, B/OX was first detected at the site in 2013. All images were taken at White Clay Creek State Park, New Castle County, Delaware.

All genotyped adults (*n* = 37) were related to at least one other bird in the sample at the cousin level or greater (*r* ≥ 0.125), and 81% were related to at least one other bird at the half-sibling level or greater (*r* ≥ 0.25, range 0.25–0.60). Twenty-four percent of *r*-values (*n* = 666) were at least as high as would be expected between cousins (*r* ≥ 0.125; Table 2). Two multi-generational genetic clusters were evident in the sample (Fig 1). One cluster included 7 females and 13 males, and the other cluster included 9 females and 8 males. Relatedness was significantly higher for within-cluster pairs (*n* = 327) than between-cluster pairs (*n* = 339; Mann Whitney *U*-test, W = 90260, *P* < 0.001). Nine SY birds (5 females, 4 males) had *r*-values indicating close kinship with each other and with ASY adults in the 2012 sample, suggesting a high probability that they had hatched at the site in 2011 and returned the following year (after their inaugural migration). Three SY pairs had *r*-values indicating probable sibling-level relationships. One nestling (B/OX) that was banded at the site in 2011 (1 of 23 banded young that survived to fledging) was captured as an adult in 2013 and 2014 (Fig 2).

**Table 2:** Summary of genetic relatedness in a sample of 37 adult Veeries (*n* = 666 pairwise combinations) from White Clay Creek State Park, New Castle County, Delaware. Data represent *r*-values for genotype pairs derived from six polymorphic microsatellite loci [33], as calculated via KINGROUP 2.0.

*r*-values	# of pairs	%
≥ 0.50	4	0.6
0.40–0.50	9	1.4
0.30–0.40	19	2.9
0.20–0.30	60	9.0
0.10–0.20	100	15.0
0–0.10	142	21.3
≤ 0	343	51.5

### Mating systems and behavior

**Fig 3:**
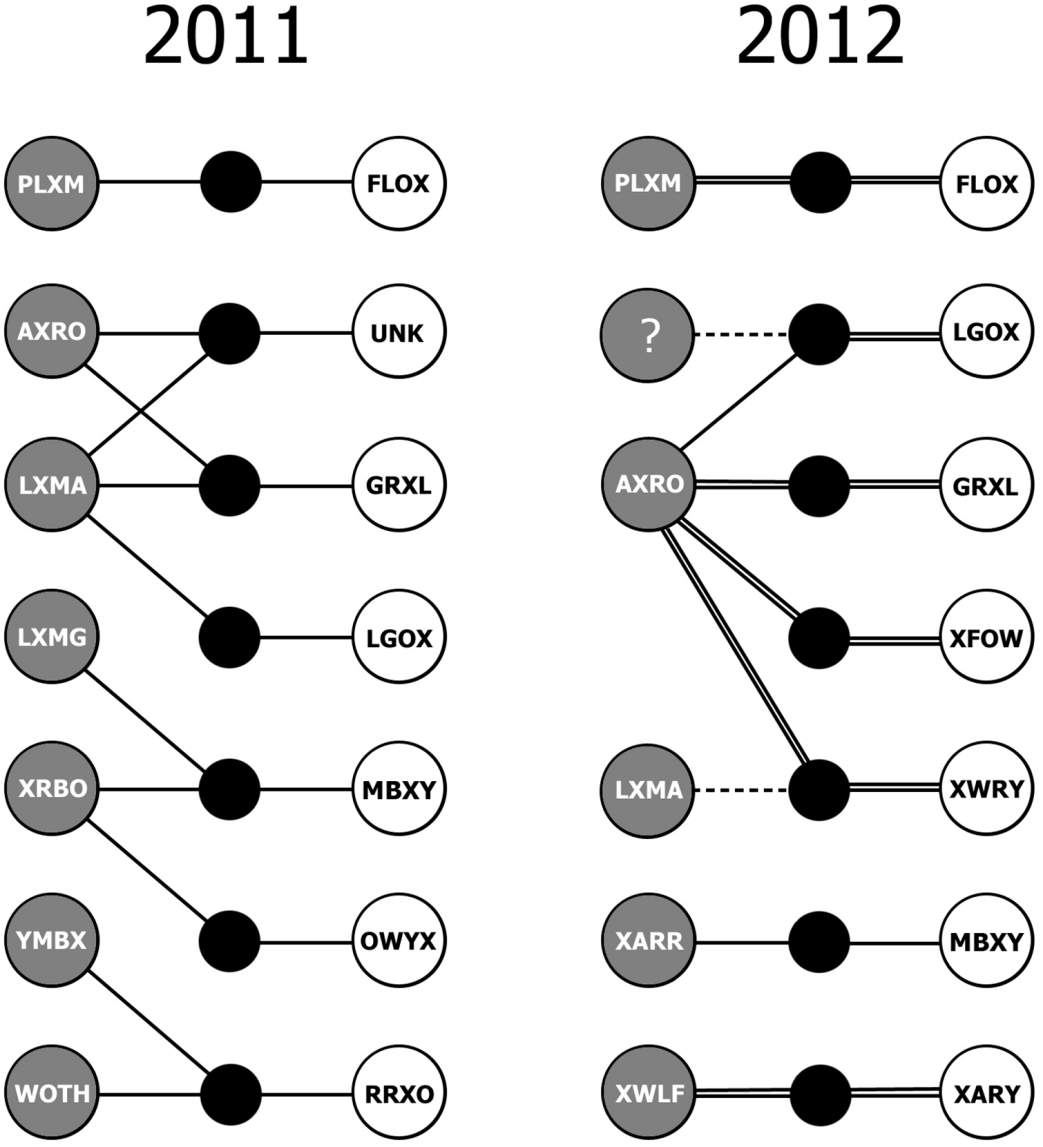
Patterns of feeding and parentage at nests of the Veery (*n* = 14) during the 2011 and 2012 breeding seasons at White Clay Creek State Park, New Castle County, Delaware. Males and females are denoted by gray and white circles, respectively. One female in 2011 was unbanded (UNK). Solid lines represent feeding relationships between adults and the nests they attended (black circles). Double solid lines denote feeding and parentage. Dashed lines represent parentage only (i.e., extra-pair fertilizations), as determined via parental exclusion. LXMA fathered one nestling in the nest attended by his close relative AXRO (*r* = 0.254) and female XWRY. An unknown father (?) sired one nestling in the nest attended by AXRO and female LGOX. The 2012 brood fed by XARR and MBXY was not genotyped. One nest in 2011 was attended by a heterospecific adult, denoted "WOTH" [45].

Fourteen nests were attended by color-banded adults and monitored with video cameras and/or direct observation (Fig 3), of which 10 were socially monogamous (71.4%), but at 60% of socially monogamous nests (6/10), the male was also detected as a feeder at a second, third, or fourth nest in the same season (each with a different female). Four females (FLOX, LGOX, GRXL, MBXY) and 3 males (PLXM, AXRO, LXMA) were detected as feeders in both years. Multiple male feeders were detected at 4 nests in 2011, and not at all in 2012 (Fig 3). All genotyped male-female Veery pairs for which nesting behavior was monitored were unrelated (*n* = 8, *r*_max_ = 0.051, mean = -0.11 ± 0.15). In the complete sample (*n* = 36 nests, including those lacking video data), 41.7% of nests fledged at least one young. All confirmed trio nests (*n* = 4) fledged ≥ 2 young. Fifty six percent of nests failed before fledging (20/36), of which half failed during the incubation phase (10/36, 27.8%) and half during the nestling phase (10/36, 27.8%). The number of young fledged per female per season ranged from 0 to 4 (2011: *n* = 13, *x̅* = 1.69 ± 1.8; 2012: *n* = 11, *x̅* = 0.82 ± 1.25), and did not differ between years (Mann Whitney *U*-test, *W* = 181.5, *P* = 0.24). All females were single brooded and second/third nesting attempts were only observed after failure. No female was known to fledge young in both years.

The frequency of multiple male feeders and multiple nests per male that we detected may be an underestimation because we may not have located every nest at the site, we were unable to film each nest that was located, and cameras were only running for a small fraction of daylight hours. PLXM (male) and FLOX (female) were apparently a socially monogamous pair in both years (Fig 3), but PLXM was seen carrying food in the opposite direction of the nest in 2011, so he may have been attending an additional nest that was not located. Multiple male feeders were detected at four of seven nests (57%) in 2011, of which two were attended by closely related males AXRO and LXMA (*r* = 0.254). A third trio nest was attended by two males of equal age (LXMG, XRBO) that did not return in 2012, and were therefore not genotyped. The fourth trio nest in 2011 was attended by two adult Veeries (male YMBX, female RRXO) and a male Wood Thrush (*Hylocichla mustelina*), which fed at a higher rate than either Veery [45]. At each trio nest, one male seemed to be the primary feeder and a second male was detected at infrequent intervals. For example, male XRBO was detected as a feeder at the nest of female MBXY a total of 12 times during 4 video sessions on June 14, 15, 17 (total duration = 19 h 43 m), during which time LXMG was detected feeding 54 times. XRBO was not detected in a 3 h video session on June 21, but LXMG and MBXY fed the young 14 and 11 times, respectively. In 2012, LXMG and XRBO were not detected at the study site, and an influx of new SY males were recruited to the population (e.g., XARR, who shared an apparently monogamous nest with MBXY; and XWLF, who paired with SY female XARY, Fig 3).

For all 6 genotyped broods in 2012, the female attending the nest was the genetic mother of the entire brood, and no female was observed feeding at more than one nest (Fig 3). Four of six broods were entirely sired by the male that fed them, and one chick in each of the remaining two nests was fathered by a different male that was not detected feeding them (Fig 3). AXRO (4 years old) fathered young in 3 of 4 nests at which he was detected feeding in 2012, of which one included a nestling fathered by his close kin LXMA (*r* = 0.254). AXRO co-fed with LXMA at the nests of two females in 2011. Three of AXRO's four females in 2012 (75%) were related at the half-sibling level (mean *r* = 0.248, range 0.195–0.275) and AXRO was observed mate guarding two of the females (GRXL and XFOW, *r* = 0.195), whose laying periods were asynchronous [32]. At another nest attended by AXRO, a closely related SY male (XGLA, *r* = 0.24; AXRO's likely half-brother, nephew, or grandson) was observed on multiple days approaching to within 2 m of the nest, exchanging soft calls with AXRO and/or XFOW, but was not detected on video as a feeder in 36 hrs of footage. When XFOW's brood was depredated on the same day that GRXL's brood hatched (June 10, 2012), AXRO was observed guarding the former female on June 13 and 18 as she prepared a second nesting attempt. Concurrently, AXRO was absent in 7 video sessions at the nest of GRXL (17.97 hr of footage, June 10–15), during which time the brood of GRXL was reduced by two, both predation events occurring in between video sessions. AXRO was eventually detected as a feeder at GRXL's nest on June 15, by which time a sufficient number of days had passed for XFOW to re-clutch [32]. When AXRO approached the nest with food, GRXL assumed a hostile "horizontal stretch" posture with bill gaping [44] for several seconds in apparent defense of the brood (Fig 2), after which she acquiesced and left the nest, and AXRO delivered food to their offspring.

## Discussion

Multi-generational kinship occurs among Veeries at our study site, among and between SY and ASY adults of both sexes. Although our sample size was modest, the genetic structure that we detected among birds of known age can only have resulted from a non-zero rate of natal philopatry over multiple years. Despite banding very few nestlings in 2011 (*n* = 34, of which 23 fledged), we recaptured one (B/OX) as an adult male in the 2013 and 2014 breeding seasons, corroborating the genetic data showing kin structure. Some SY males and females had *r*-values indicating sibling-level relationships with other SY males and females; and they were closely related to ASY adults in their respective genetic clusters. This implies that the kin structure is maintained by natal philopatry in both sexes, not just in males. This result was unexpected because annual movements of adult Veeries that breed at our study site regularly exceed 12,000 km and include a mid-winter intratropical migration within South America [26,27]. Kinship among adults on the breeding grounds of migratory passerines is generally assumed to be uncommon because natal philopatry contributes to inbreeding depression, and it is not expected to be an evolutionary cause of polygynandry [36,46,47].

Multiple mating was detected in both sexes and all "helpers" were breeding males with a potential stake in the paternity of the broods they fed, as in Bicknell’s Thrush, Dunnock, Smith's Longspur (*Calcarius pictus*), and other polygynandrous passerines for which cooperative parental care is not expected to be influenced by kin selection [5,19,21,48]. Notwithstanding the kin structure among adult Veeries at our site, we detected no evidence of matings between close genetic relatives (*r* ≥ 0.25), as have been reported in some polygynandrous species (e.g., Pukeko, *Porphyrio porphyrio*) [49]. Male Veeries in our sample did not sire offspring with nor attend the nests of closely related females. In 2013, after the conclusion of the present study, a nesting pair was found that was related at the cousin-level (*r* = 0.184; C. M. Heckscher unpub. data), but for all genotyped nesting pairs and trios in the 2011–2012 sample, the male(s) were unrelated to the female.

We detected four modes of parental care in our sample that varied within and between years, including multiple male feeders that attended a single brood, males that concurrently attended multiple broods that were not attended by other males, two males that co-attended the same two broods, and apparently socially monogamous pairs. These patterns were not unlike those described in Bicknell's Thrush and other polygynandrous species [8,19,21], except that some Veeries at our site apparently interact with close genetic relatives in successive breeding seasons over multiple years. Cooperative parental care was also recently documented in Slaty-backed Nightingale-Thrush (*Catharus fuscater*) [50], a sedentary Neotropical resident from a relatively distant clade in the *Catharus* phylogeny [51], casting doubt on the hypothesis that the flexible mating systems of the Veery and Bicknell's Thrush are a derived feature of the clade they share with Gray-cheeked Thrush (*Catharus minimus*). Studies of color-banded individuals are lacking for most *Catharus* species, precluding further assessment of phylogenetic causes at this time.

Observed patterns of parental care in our small sample of Veery nests matched those that would be expected if each individual acted so as to maximize its inclusive fitness [13,14]. Two closely related males (*r* = 0.254) shared two nests (with two different females) in 2011, and in successive years, three closely related females were mated to those males. We have no evidence that social interaction among kin extends beyond close relatives that nest in proximity, but the kin structure may have a broader effect on social behavior if closely related males compete with each other for access to unrelated females, and if intersexual conflict occurs most frequently between non-relatives [3]. It is not uncommon, in other polygamous species with fine-scale genetic structure, to find that closely related co-breeders of one sex are unrelated to the breeder of the opposite sex [52,53]. Future research should address whether and how Veeries discriminate against kin during mate selection. Kin recognition has been demonstrated in a large number of cooperative and non-cooperative bird societies and, although not theoretically necessary for kin selection to operate [14,54], presumably enables individuals to maximize inclusive fitness by making facultative adjustments to their parental investment as the breeding season progresses [55–57]. Kin recognition may also function as an inbreeding avoidance mechanism in migratory species that exhibit natal philopatry in both sexes, like Savannah Sparrows (*Passerculus sandwichensis*) [36], and in opportunistically polygynandrous species that live in groups of genetically related individuals, like Acorn Woodpeckers (*Melanerpes formicivorus*) [58].

Female Veeries at our study site are typically single brooded and produce fewer than four offspring per year (nests with five eggs are uncommon and rarely, if ever, fledge five young). Because of this apparent limitation, males that monopolize multiple females can produce three or four times more offspring each year than monogamous males. Therefore, we hypothesize that females will be disproportionately attracted to polygynous males despite the risk of mate desertion because "faithful" males may carry lower quality genes (i.e., those that support phenotypes unlikely to achieve polygyny) [3,4]. Female Veeries are not gregarious enough to be physically guarded as a group, but male Veeries can achieve polygyny by guarding multiple females with asynchronous fertile periods, and/or excluding rival males from the vicinity of females and their nests [32,59]. The prevalence of each strategy may fluctuate as social and ecological conditions change, as has been shown to occur in other thrush species [60,61]. Heckscher [59] found that some male Veeries use song, calls, and postural displays to defend large overlapping "territories", but tolerate the nests of "covert" males (i.e., non-territorial) within their territories while maintaining nests of their own and continuing to defend against adjacent territorial males. These patterns of behavior, and the extent of territory overlap more generally, may be related to the kin structure if unrelated males do not compete for the same females, or they may reflect dominance hierarchies among related males [32].

Multiple mating by female Veeries may be a compensatory response to polygyny in males, which results from the differential cost of gamete production. Females that attract (via multiple mating) multiple male feeders to their nests mitigate the risk of withdrawn parental care [3]. This hypothesis has sometimes been neglected in favor of explanations involving direct benefits conferred to the female (e.g., "nuptial gifts" from males), genetic compatibility between mates, genetic benefits conferred to offspring, and/or benefits of having genetically diverse progeny [5]. We are unable to reject all of these hypotheses, but our data show that polygynous male Veeries may cease feeding at nests containing their own genetic progeny if an additional female to whom they are paired becomes fertile and must be guarded [32]. Dunnocks make the same "choice" in that scenario [21]. Also, male Veeries have not been detected feeding females during the incubation period (i.e., no nuptial gifts despite female-only incubation), and in rare instances, multiple mating by female Veeries may even entice heterospecific adults to the nest, a scenario in which parental care is augmented without conferring any genetic benefits [45].

When the optimal strategies of the sexes are inherently conflicted, few individuals are expected to achieve the best outcome each year, and multiple mating by females, if it attracts multiple male feeders to the nest, may be an effective counter strategy that buffers against the risk of mate desertion while potentially increasing parental care and/or nest defense [3]. Females may be most successful in attracting multiple male feeders if they settle near males that have roughly equivalent competitive ability and overlapping home ranges, and near non-hostile females. Unlike female Veeries, female Dunnocks are commonly double brooded, occupy non-overlapping home ranges, and when nesting in polygynandrous combinations frequently destroy each other's eggs. Because of this intrasexual "interference", female Dunnocks fare no better in polygynandry than in polygyny even though they gain the parental support of an additional male [21]. Egg destruction is also common among co-breeder females in the polygynandrous, communally nesting Acorn Woodpecker [58]. By contrast, we observed no territorial hostility among related female Veeries that nested in proximity. We hypothesize that female Veeries that tolerate the nests of female kin near their own would stand to maximize their inclusive fitness, if doing so decreased the probability of brood failure for each female. Similarly, related male Veeries with overlapping home ranges may be more likely to tolerate each other as co-feeders, and invest at polyandrous nests where their paternity is uncertain, if doing so optimizes their inclusive fitness [14]. Like Acorn Woodpeckers, parental investment of male Veeries may be greater at nests where they stand to gain a direct benefit via parentage, coupled with the indirect benefits of assisting kin [58].

It is not yet known whether variability in modes of parental care in the Veery are proximately influenced by a limiting ecological resource, but this is inferred to be the case because it has been demonstrated or inferred in many polygynandrous bird species with or without non-breeding kin helpers [15,21,24,58]. Nevertheless, the kin structure among adults at our study site is presumably costly to maintain because of the entropic influence of trans-hemispheric migration and the potential for inbreeding (which may be minimal if Veeries recognize kin). Inclusive benefits may be sufficient for the mechanism by which the kin structure is maintained (i.e., natal philopatry) to be favored by natural selection. Our results indicate that kin selection should not be ruled out *a priori* as a potential factor affecting mating system evolution of migratory songbirds. Future research of the mating systems of the Veery should aim to identify and test whether and how mating strategies are affected by the scarcity of ecological resources, and how the strength of competition for those resources varies with kin structure. We expect the occurrence and extent of multi-generational kinship to fluctuate with breeding density and rates of adult longevity, natal return, and immigration. If the frequency of cooperative parental care is dependent upon the kin structure, we expect it to wax and wane correspondingly.

## Supporting Information

S1 File Raw data from fragment analysis. This compressed file (.zip) includes the raw data files from the fragment analysis of PCR products from the 6 microsatellite markers used in this study. Two folders contain data files (.fsa) for two multiplexed samples, the first containing loci Cuμ02 (tagged with 6FAM), Cuμ04 (VIC), Cuμ05 (NED), and the second containing loci Cuμ10 (6FAM), Cuμ28 (VIC), and Cuμ32 (NED). Each bird in the sample (*n* = 58, including 37 adults and 21 offspring) is denoted with an odd number (1–115), and the file names correspond to those numbers (see S2 File).
(ZIP)

S2 File Sample allele distributions. Excel spreadsheet with distributions of alleles at 6 microsatellite loci used in this study, from a sample of 37 adult Veeries and 21 offspring at White Clay Creek State Park, DE.
(XLSX)

S3 File Genetic relatedness values. KINGROUP 2.0 output showing individual genotypes and pairwise *r*-values (*n* = 666) for a sample of 37 adult Veeries at White Clay Creek State Park, DE.
(CSV)
